# Complement C5a-C5aR1 signalling drives skeletal muscle macrophage recruitment in the hSOD1^G93A^ mouse model of amyotrophic lateral sclerosis

**DOI:** 10.1186/s13395-017-0128-8

**Published:** 2017-06-01

**Authors:** Haitao A. Wang, John D. Lee, Kah Meng Lee, Trent M. Woodruff, Peter G. Noakes

**Affiliations:** 1 School of Biomedical Sciences, The University of Queensland, St Lucia, QLD 4072 Australia; 2 University of Queensland Centre for Clinical Research, The University of Queensland, Herston, QLD 4029 Australia; 3 Queensland Brain Institute, The University of Queensland, St Lucia, QLD 4072 Australia

**Keywords:** Complement, C5, C5aR1, Inflammation, Macrophages, Skeletal muscle

## Abstract

**Background:**

The terminal pathway of the innate immune complement system is implicated in the pathogenesis of amyotrophic lateral sclerosis (ALS). Terminal complement activation leads to generation of C5a, which through its receptor, C5aR1, drives immune cell recruitment and activation. Importantly, genetic or pharmacological blockage of C5aR1 improves motor performance and reduces disease pathology in hSOD1^G93A^ rodent models of ALS. In this study, we aimed to explore the potential mechanisms of C5aR1-mediated pathology in hSOD1^G93A^ mice by examining their skeletal muscles.

**Results:**

We found elevated levels of C1qB, C4, fB, C3, C5a, and C5aR1 in tibialis anterior muscles of hSOD1^G93A^ mice, which increased with disease progression. Macrophage cell numbers also progressively increased in hSOD1^G93A^ muscles in line with disease progression. Immuno-localisation demonstrated that C5aR1 was expressed predominantly on macrophages within hSOD1^G93A^ skeletal muscles. Notably, hSOD1^G93A^ × C5aR1^-/-^ mice showed markedly decreased numbers of infiltrating macrophages, along with reduced neuromuscular denervation and improved grip strength in hind limb skeletal muscles, when compared to hSOD1^G93A^ mice.

**Conclusion:**

These results indicate that terminal complement activation and C5a production occur in skeletal muscle tissue of hSOD1^G93A^ mice, and that C5a-C5aR1 signalling contributes to the recruitment of macrophages that may accelerate muscle denervation in these ALS mice.

## Background

Amyotrophic lateral sclerosis (ALS) is a late-onset fatal neurodegenerative disease that is characterized by muscular weakness and paralysis, ultimately leading to death typically within 2 to 5 years. The disease involves the progressive degeneration of motor neurons in the central nervous system (CNS) and denervation of neuromuscular synapses in the peripheral nervous system. The precise underlying causes and pathogenesis of ALS are still unknown. However, accumulating evidence indicates that there is a significant contribution of immune and inflammatory responses that accompany motor neuron degeneration [1]. These include the activation of astrocytes and microglia, and T cell and monocyte infiltration in the CNS and skeletal ﻿muscles﻿ of ALS patients and animal models [2–6].

The complement system is a key component of the immune system, and has long been implicated in the pathogenesis of ALS. Numerous clinical and animal studies have demonstrated strong up-regulation of activation fragments of complement components C1q, C3, and C4 in the serum, cerebrospinal fluid and neurological tissue (including spinal cord and motor cortex) of ALS patients and animal models of ALS [7–9]. Despite this strong evidence of early complement factor upregulation in ALS, specific genetic deletion of C1q, C3, and C4 in hSOD1 transgenic mouse models does not confer any beneficial effects on disease progression [9, 10]. These results contrast with our prior work demonstrating that genetic deletion, or pharmacological inhibition, of C5aR1, is neuroprotective in hSOD1 transgenic rodent models of ALS [11–13]. This suggests that activation of complement at the downstream step of C5 (i.e., the terminal pathway which generates C5a) may be the key point at which complement-mediated neurotoxicity occurs in these ALS models [8, 13]. The precise mechanisms by which complement activation and C5a-C5aR1 signalling are driving neurodegeneration in ALS mice is still unknown.

In addition to CNS activation of complement, there is evidence of complement activation within the peripheral nervous system of ALS. It is well established that the degeneration of motor axons in the periphery is an early and significant pathological feature in ALS patients and hSOD1 transgenic mice [14, 15]. Complement components are expressed within the peripheral nervous system [16] and C3 activation products, C1q and C4, are deposited on the denervated and degenerated neuromuscular junctions in hSOD1^G93A^ mice [10, 17]. However, to date, there is no information regarding the expression of terminal complement components in hSOD1^G93A^ mice.

In the present study, we therefore examined the expression of major complement factors, and of C5a and its receptor C5aR1, in tibialis anterior (TA) muscles of wild-type (WT) and hSOD1^G93A^ mice at defined disease stages. We found that C5a and C5aR1 were up-regulated, and that C5aR1 was expressed by infiltrating CD11b^+^ macrophages. Interestingly, hSOD1^G93A^ mice lacking C5aR1 showed dramatic reductions in infiltrating macrophages in TA muscles, along with reduced neuromuscluar denervation and improved muscle strength. These results demonstrate that activation of complement, specifically C5a-C5aR1 signalling, may be involved in the disease progression of hSOD1^G93A^ mice, through limiting infiltration of peripheral macrophages into degenerating muscles.

## Methods

### Mice

Transgenic hSOD1^G93A^ mice were obtained from Jackson Laboratory (Bar Harbor, ME, USA) and were bred on C57BL/6J background to produce hSOD1^G93A^ mice and WT control mice. These hSOD1^G93A^ mice carry a high copy number of the mutated allele of the human SOD1 gene [18]. Homozygous C5aR1 deficient mice (C5aR1^-/-^) were kindly provided by Dr. Rick Wetsel and described previously [19]. To generate hSOD1^G93A^ mice lacking C5aR1 (hSOD1^G93A^ × C5aR1^-/-^), transgenic heterozygous hSOD1^G93A^ male were first cross-bred with C5aR1^-/-^ females to generate F1 progeny (hSOD1^G93A^ × C5aR1^+/-^). hSOD1^G93A^ × C5aR1^+/-^ males were then cross bred with C5aR1^-/-^ females to obtain F2 progeny (hSOD1^G93A^ × C5aR1^-/-^). Female WT, hSOD1^G93A^, C5aR1^-/-^ and hSOD1^G93A^ × C5aR1^-/-^ mice at three pre-defined stages were used in this study, as described previously [8].

### Real-time quantitative PCR

Total RNA was isolated from TA muscle of WT and hSOD1^G93A^ mice using RNeasy Lipid Tissue extraction kit (QIAGEN Inc., Alameda, CA, USA) per the manufacturer’s protocol. The total RNA was purified from genomic DNA contamination using Turbo DNAse treatment (Ambion, Life Technologies, Carlsbad, CA, USA) then converted to cDNA by means of a reverse transcription kit (Agilent Technologies Inc., Santa Clara, CA, USA) per the manufacturer’s protocol. Commercially available gene-specific Taqman probes (Applied Biosystems, Life Technologies, Carlsbad, CA, USA) were used to amplify target gene of interest as described previously [8]. Relative target gene expression to glyceraldehyde-3-phosphate dehydrogenase (GAPDH) was determined using this formula: 2^-∆CT^ where ∆Ct = (Ct target gene – Ct GAPDH) [20]. Final measures are presented as relative levels of gene expression in hSOD1^G93A^ mice compared with expression in WT controls.

### Western Blot analysis

TA and soleus (SOL) muscle homogenates from WT and hSOD1^G93A^ mice at different disease stages were resolved on a 10% SDS-PAGE gel and transferred to nitrocellulose membranes (Pall Corporation, Cheltenham, VIC, Australia). Membranes were blocked with 2.5% skim milk in Tris-buffered saline-Tween (TBST) solution (containing 1× TBS and 0.1% Tween 20) for 1 h at room temperature and were subsequently incubated with anti-C5aR1 antibody overnight at 4 °C (1:2,500 dilution in 2.5% SM-TBST; BMA Biomedical, Augst, Switzerland). Membranes were washed 3 × 10 min with TBST and then incubated with the goat anti-chicken horseradish peroxidase (HRP; 1:15,000 dilution in 2.5% SM-TBST, GE Healthcare, Pittsburgh, PA, USA) for 1 h at room temperature. After a final wash with TBST for 6 × 5 min, signals were detected using the ECL system (GE Healthcare, Pittsburgh, PA, USA). Blots were then stripped and reprobed with anti-GAPDH (1:15,000; Millipore, Billerica, MA, USA) and then detected with sheep anti-mouse HRP (1:4,000; GE Healthcare, Pittsburgh, PA, USA) as loading control. Semi-quantitative densitometric analysis of these immunoreactive bands was carried out to determine differences in C5aR1 expression levels between WT and hSOD1^G93A^ TA and SOL muscle samples at different disease stages as described previously [8].

### Enzyme-linked immunosorbent assay

Ninety-six well-plate (Greiner Bio-One, Frickenhausen, Germany) was pre-coated with monoclonal rat anti-mouse C5a capture antibody (Clone I52 – 1486; BD Pharmingen, San Diego, CA, USA) diluted in coating buffer (100μM, NaHCO_3_, 34μM Na_2_CO_3_, pH 9.5) overnight at 4 °C in a sealed humidified container. This capture antibody is specific for a neo-epitope exposed only in mouse C5a/C5a desArg and does not cross-react with C5 [8]. Following the plate being blocked for 1 h at room temperature with assay diluent (10% FCS/PBS), C5a standard and TA muscle homogenates was incubated for 2 h at room temperature. The plates were subsequently incubated with biotinylated rat anti-mouse C5a detection antibody (clone I52-278; BD Pharmingen, San Diego, CA, USA) for 1 h at room temperature, and then incubated with Streptavidin-HRP conjugate for 30 min at room temperature. Tetramethylbenzidine (Sigma-Aldrich, Saint Louis, MO, USA) substrate was used as the chromogen and the plate was read at 450 nm. Levels of C5a in TA muscle samples were adjusted to micrograms per protein and expressed as nanograms of C5a per microgram of protein.

### Immunohistochemistry

Transverse cryosections (10 μm) from the TA muscles of WT, hSOD1^G93A^, C5aR1^-/-^ and hSOD1^G93A^ × C5aR1^-/-^ mice were stained to localise the expression of C5aR1 with specific cell-type markers for neuromuscular junctions (alpha-Bungaratoxin (αBTX), 1:5000, Invitrogen, Eugene, OR, USA), Schwann cells (rabbit S100β, 1:1000, DAKO, Osaka, Japan), and macrophages (rat CD11b, 1:250, Abcam, Cambridge, MA, USA). Briefly, the sections were blocked in phosphate-buffer saline (PBS) containing 2% normal goat serum (Sigma-Aldrich, Saint Louis, MO, USA) and 0.2% Triton X-100 (Sigma-Aldrich, Saint Louis, MO, USA) at room temperature for 35 min and incubated with primary antibodies at 4 °C overnight. After incubation, the sections were washed in PBS and then incubated with an appropriate Alexa conjugated secondary cocktail at room temperature for 2 h: Alexa 555 donkey anti-rat (1:1000), Alexa 488 donkey anti-rat (1:600), and Alexa 488 goat anti-rabbit (1:600, Invitrogen, Life Technologies, Mulgrave, VIC, Australia). All the primary and secondary antibodies were diluted in PBS with 2% bovine serum albumin and 0.2% Triton X-100. All sections were incubated with 4,6-diamidino-2-phenylindole (DAPI; Invitrogen, Life Technologies, Mulgrave, VIC, Australia) for 5 min at room temperature prior to being mounted with Prolong Gold Anti-Fade medium (Invitrogen, Life Technologies, Mulgrave, VIC, Australia). Fluorescent signals were observed using a Zeiss LSM Meta 510 upright confocal microscope with a Plan-Apochromat 63× oil objective (Carl Zeiss Inc., Oberkochen, Germany).

For the neuromuscular junction (NMJ) denervation analysis, transverse cryosections (10 μm) from TA muscles of WT, hSOD1^G93A^, C5aR1^-/-^ and hSOD1^G93A^ × C5aR1^-/-^ mice at mid-symptomatic stage were also stained to determine denervation status of these animals. In brief, sections were blocked with 2% bovine serum albumin, 2% normal goat serum and 0.1% Triton X-100 for 30 min followed by incubation with primary antibody (rabbit anti-Synaptophysin (SNP, 1:250, Sigma Aldrich, Saint Louis, MO, USA) overnight at 4 °C. After incubation with primary antibody, sections were incubated in combination of secondary antibody of Alexa 488 goat anti-rabbit (1:1000) and Alexa 555 αBTX (1:5000, Invitrogen, Life Technologies, Mulgrave, VIC, Australia) for 2 h at room temperature. All the primary and secondary antibodies were diluted in PBS with 2% bovine serum albumin and 0.1% Triton X-100. Sections were then washed with PBS prior to being mounted with Prolong Gold Anti-Fade medium (Invitrogen, Life Technologies, Mulgrave, VIC, Australia). Sections were imaged with an Olympus Fluoview FV1000 confocal laser scanning microscope using a 40×/0.95 NA Uplan-Apochromat objective.

### Quantification of peripheral macrophages

TA muscle sections were stained for macrophages (CD11b and CD68) in WT, hSOD1^G93A^, C5aR1^-/-^ and hSOD1^G93A^ × C5aR1^-/-^ mice (10 sections spaced 100 μm apart per animal, *n* = 3). With a 20× objective, five random regions (874 × 655 × 10 μm) from each section were selected without any knowledge of the presence of positive cells by viewing only in DAPI channel. Each selected region was imaged with standardized settings and then saved. All cell profiles with a clear fluorescently labelled membrane that were within a single plane of focus were counted, and the average number of cells of interest from 50 selected regions for each animal was used for statistical analysis.

### Quantification of NMJ denervation

Each image was captured using identical laser power levels, photomultiplier gain levels, scanning speed, and pinhole size amongst different slides using the same antibody. Specific criteria were applied for scoring of innervation status of NMJs across all genotypes. Endplates were considered innervated when the postsynaptic endplates displayed co-localisation with the presynaptic marker (SNP). Denervated endplates were counted when no co-localisation of presynaptic marker was observed in relation to the postsynaptic AChRs.

### Hind-limb grip strength test

A digital force gauge (Ugo Basile) was used to measure maximal hind-limb muscle grip strength as described previously [8, 11]. In brief, mice were held by their tail and lowered until their hind limbs grasped the T-bar connected to the digital force gauge. The tail was then lowered until the body was horizontal with the apparatus, and mice pulled away from the T-bar with a smooth steady motion until both of their hind limbs released the bar. The strength of the grip was measured in gram force. Each mouse was given ten attempts and the maximum grip strength from these attempts recorded. The mouse genotypes were not made available to the researcher (JDL) conducting the hind-limb grip strength test.

### Statistical analysis

All measures were performed using GraphPad Prism 7.0 (GraphPad Software Inc., San Diego, CA, USA). For the results from quantitative real time PCR, western blotting and enzyme-linked immunosorbent assay, statistical differences between WT and hSOD1^G93A^ mice were analysed using two-tailed Student *t*-test at each stage of disease progression. The statistical differences between WT, hSOD1^G93A^, C5aR1^-/-^, and hSOD1^G93A^ × C5aR1^-/-^ mice for peripheral immune cell numbers, NMJ denervation status and hind-limb grip strength were analysed using one-way ANOVA with Tukey’s *post hoc* test for each stage of disease progression. All data are presented as mean ± SEM and differences were considered significant when *p* ≤ 0.05.

## Results

### Dysregulation of complement system in the tibialis anterior muscle of hSOD1^G93A^ mice

Previous studies, including our own, have shown up-regulation of major complement components in the lumbar spinal cord and skeletal muscle of hSOD1^G93A^ mice. However, there is no comprehensive overview of which complement pathway is dysregulated in the skeletal muscle of hSOD1^G93A^ mice during disease progression. To investigate this, we measured the mRNA levels of initiating components of the classical/lectin pathway (C1qB and C4), alternative pathway (factor B; fB), the central component to all pathways (C3) and the complement regulators (CD55 and CD59a) in the TA muscle of WT and hSOD1^G93A^ mice, respectively, using quantitative real-time PCR. The TA muscle of hSOD1^G93A^ mice was chosen for this study as it consists of fast-twitch muscle fibres that are preferentially affected in these animals when compared to slow-twitch muscle fibres [21, 22].

**Fig. 1:**
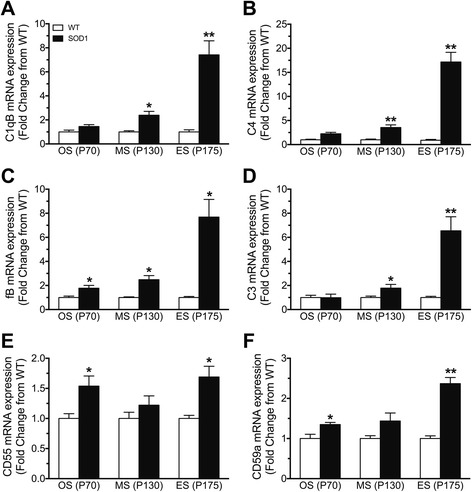
Dysregulation of complement components in tibialis anterior muscle (TA) of hSOD1^G93A^ mice at three different stages of disease progression. Panels **a** to **f** show the mRNA expression profiles of the following complement components: C1qB (**a**, classical pathway), C4 (**b**, classical/lectin pathway), fB (**c**, alternative pathway), C3 (**d**, central component), CD55 (**e**, regulator), and CD59a (**f**, regulator) in TA muscle of hSOD1^G93A^ mice (SOD1, *black bars*) relative to wild-type (WT, *white bars*) mice during onset (OS; postnatal day 70 (P70)), mid-symptomatic (MS; postnatal day 130 (P130)) and end-stage of disease (ES; postnatal day 175 (P175)). Data are expressed as means ± SEM (*n* = 5 mice/group, * *p* < 0.05, ** *p* < 0.01, Student *t* test 6)

Quantitative real-time PCR analysis showed significant increases in C1qB and C4 transcripts by 2.4-fold and 3.5-fold at mid-symptomatic stage and 7.4-fold and 17.2-fold at end-stage of disease when compared to WT mice, respectively (*n* = 5, *p < 0.05 and **p < 0.01; Fig. 1a, b). In addition to C1qB, fB also displayed a marked increase in mRNA levels by 1.8-fold at onset stage, 2.5-fold increase at mid-symptomatic and 7.7-fold increase at end-stage of disease (*n* = 5, *p < 0.05; Fig. 1c). The central component of complement system C3 was also increased in TA muscle of hSOD1^G93A^ mice. Specifically, we observed 1.8-fold and 6.5-fold increases at mid-symptomatic and end stage of disease when compared with WT mice (*n* = 5, *p < 0.05 and **p < 0.01; Fig. 1d).

The negative regulators of the complement system CD55 and CD59a were also investigated. CD55 and CD59a mRNA expression was increased by 1.5-fold and 1.3-fold at onset stage and 1.7-fold and 2.4-fold at end stage of disease in hSOD1^G93A^ mice when compared with WT mice, respectively (*n* = 5, *p < 0.05 and **p < 0.01; Fig. 1e, f). These results suggest widespread complement system perturbation in the TA muscles of hSOD1^G93A^ mice, which accelerates as the disease worsens.

### C5a ligand and its receptor C5aR1 are up-regulated in the tibialis anterior muscle of hSOD1^G93A^ mice

**Fig. 2:**
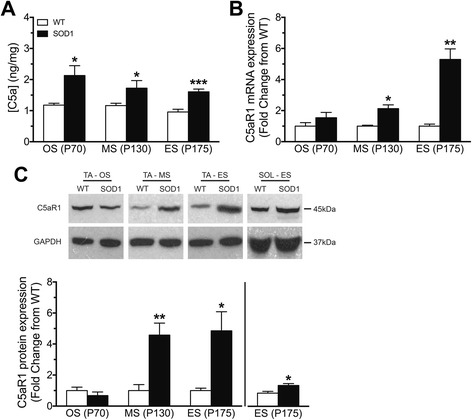
Expression of C5a and C5aR1 in tibialis anterior (TA) muscle of wild-type (WT) and hSOD1^G93A^ (SOD1) mice at three different stages of disease progression. **a** shows the protein expression of C5a in the TA muscle of WT (*white bars*) and SOD1 (*black bars*) mice at onset (OS; postnatal day 70 (P70)), mid-symptomatic (MS; postnatal day 130 (P130)) and end-stage of disease (ES; postnatal day 175 (P175). **b** shows mRNA expression of C5aR1 in the TA muscle of SOD1 mice relative to age matched WT mice at three different ages. Top panel in **c** shows a representative western blot of C5aR1 with GAPDH in the TA muscle of SOD1 mice relative to age matched WT mice at three different ages and soleus (SOL) muscle of SOD1 mice relative to age matched WT mice at end stage of disease. Bottom panel in **c** shows protein expression of C5aR1 determined by semi-quantitative densitometry in the TA muscle of SOD1 mice relative to age matched WT mice at three different ages and SOL muscle of SOD1 mice relative to age matched WT mice at end stage of disease. Data in **a**–**c** are expressed as means ± SEM (*n* = 6 mice/group; * *p* < 0.05, ** *p* < 0.01, *** *p* < 0.001, Student *t* test at each stage)

C5a, the ligand for C5aR1, is an activation fragment of the terminal complement cascade that is rapidly generated following complement cascade initiation [23]. We therefore examined the protein levels of C5a in the TA muscle of hSOD1^G93A^ and WT mice using enzyme-linked immunosorbent assay, as a biomarker for terminal complement activation. The results showed increases in C5a at onset, mid-symptomatic and end-stage of disease by 1.8-fold, 1.5-fold and 1.7-fold when compared with WT mice, respectively (*n* = 6, *p < 0.05 and ***p < 0.001; Fig. 2a). Previous studies have shown increases in C5aR1 expression in the CNS of multiple rodent models of ALS [8, 12, 24]. In this study, C5aR1 mRNA expression was significantly increased by 2.1-fold at mid-symptomatic stage and by 5.3-fold at end-stage of disease, respectively, when compared to WT mice (*n* = 5, *p < 0.05 and **p < 0.01; Fig. 2b). This change in mRNA expression was confirmed at protein level using western blot analysis, where a 45kDA C5aR1 immuno-reactive band was observed in WT and hSOD1^G93A^ mice at all stages (Fig. 2c, top panel). Semi-quantitative analyses of these bands relative to GAPDH loading control showed increased C5aR1 protein levels in the TA muscle of hSOD1^G93A^ mice by 4.6–fold at mid-symptomatic stage and by 4.9-fold at end-stage of disease when compared to WT mice (*n* = 6, * *p* < 0.05, ** *p* < 0.01; Fig. 2c, bottom panel). The expression of C5aR1 protein level was also slightly increased in SOL muscle of hSOD1^G93A^ mice by 1.6-fold at end-stage of disease when compared to WT mice (*n* = 6, * *p* < 0.05; Fig. 2c, bottom panel). Together, the results above suggest that activation of downstream factors of complement cascade, C5a and its receptor C5aR1, occurs in the TA and SOL muscle of hSOD1^G93A^ mice.

### C5aR1 is localised to macrophages but not at end-plates nor Schwann cells in hSOD1^G93A^ mice

To investigate the cellular localisation of C5aR1 that has contributed to the increased expression in TA muscle of hSOD1^G93A^ mice, we next performed immunohistochemistry for C5aR1 on TA muscle from hSOD1^G93A^ and WT mice. Transverse sections of TA muscles at end-stage of disease were immuno-stained for C5aR1 with specific cellular markers to identify motor endplate (αBTX), Schwann cells (anti-S100β), and macrophages (anti-CD11b).

**Fig. 3:**
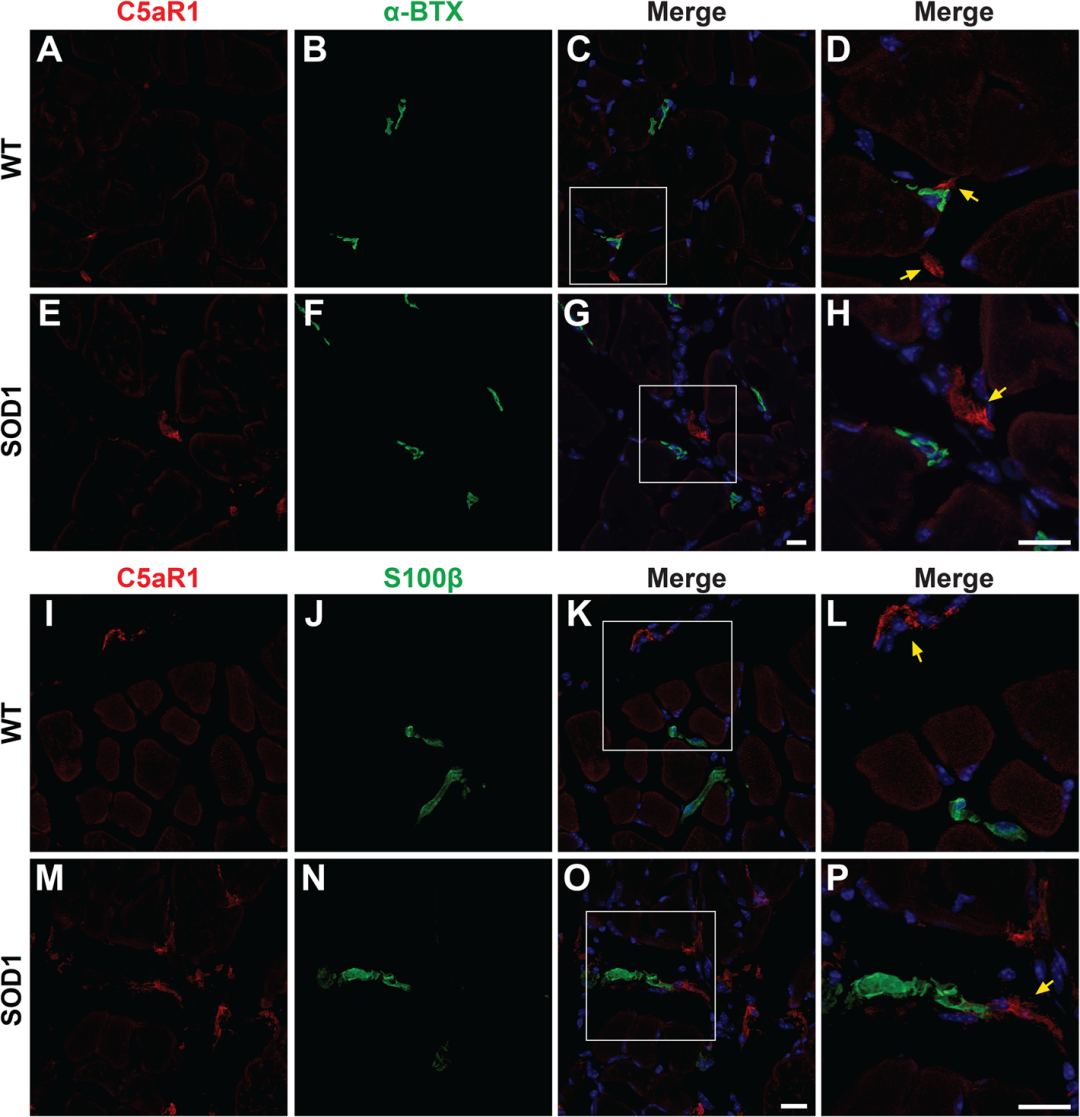
C5aR1 is not localised at the neuromuscular junction or Schwann cells in the tibialis anterior (TA) muscle of wild-type (WT) and hSOD1^G93A^ (SOD1) transgenic mice. Panels **a** to **p** shows double immuno-labelling of C5aR1 (*red*) with cellular markers (*green*) for neuromuscular junction ($\boldsymbol{\upalpha}$BTX; **a** to **c** for WT (detailed in **d**); **e** to **g** for SOD1 mice (detailed in **h**)) and Schwann cells (S100β; **i** to **k** for WT (detailed in **l**); **m** to **o** for SOD1 mice (detailed in **p**)) in the TA muscle of WT and SOD1 mice at end-stage of disease. C5aR1 is not localised to neuromuscular junction (*yellow arrows*; **d** and **h**) and Schwann cells (*yellow arrows*; **l** and **p**) as indicated by lack of co-localisation with $\boldsymbol{\upalpha}$BTX and S100β. Scale bar for all panels = 20 μm

**Fig. 4:**
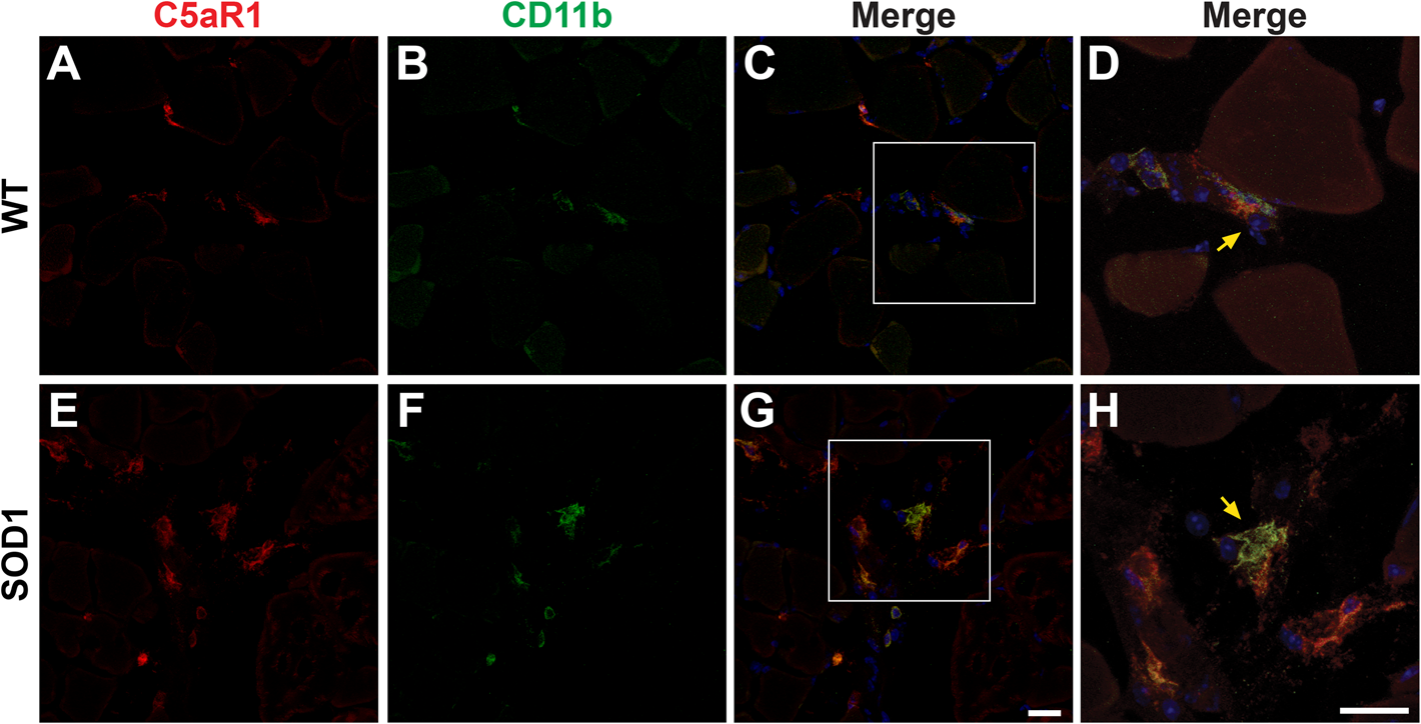
C5aR1 is localised to infiltrating CD11b^+^ macrophages in the tibialis anterior (TA) muscle of wild-type (WT) and hSOD1^G93A^ (SOD1) transgenic mice. Panels **a** to **h** show double immuno-labelling of C5aR1 (*red*) with cellular marker (*green*) for macrophages (CD11b; **a** to **c** for WT (detailed in **d**); **e** to **h** for SOD1 mice) in the TA muscle of WT and SOD1 mice at end stage disease. C5aR1 was co-localised with CD11b positive macrophages in WT and SOD1 mice (*yellow arrows* in **d** and **h**). Scale bar = 20 μm

Interestingly, we demonstrated that in WT and hSOD1^G93A^ mice, C5aR1 was not present on either motor endplate (Fig. 3a–h, and yellow arrows in corresponding merged panels 3d and 3h) or Schwann cells (Fig. 3i to p, and yellow arrows in corresponding merged panels 3l and 3p), suggesting that increased C5aR1 expression was not due to its expression at the neuromuscular junction. Following this demonstration, we then examined whether immune cells in TA muscle were responsible for the increased expression of C5aR1. We observed that C5aR1 was expressed predominantly on CD11b-positive macrophages in both WT (95.6 ± 2.2%) and hSOD1^G93A^ mice (94.1 ± 1.9; Fig. 4a–h and, yellow arrows in corresponding merged panels 4d and 4h).

### hSOD1^G93A^ mice lacking C5aR1 have reduced numbers of macrophages in the tibialis anterior muscle when compared to hSOD1^G93A^ mice

Given C5a’s role as a potent macrophage chemoattractant [25] and the presence of C5aR1-positive macrophages observed in TA muscles of hSOD1^G93A^ mice, we next investigated whether absence of C5aR1 in hSOD1^G93A^ mice impacted on peripheral macrophages infiltration. Transverse TA muscle sections of WT, hSOD1^G93A^, C5aR1^-/-^, and hSOD1^G93A^ × C5aR1^-/-^ mice at onset, mid-symptomatic and end-stage of disease progression were stained for markers of macrophages (anti-CD11b and anti-CD68) and were stereologically quantified by a blinded examiner.

**Fig. 5:**
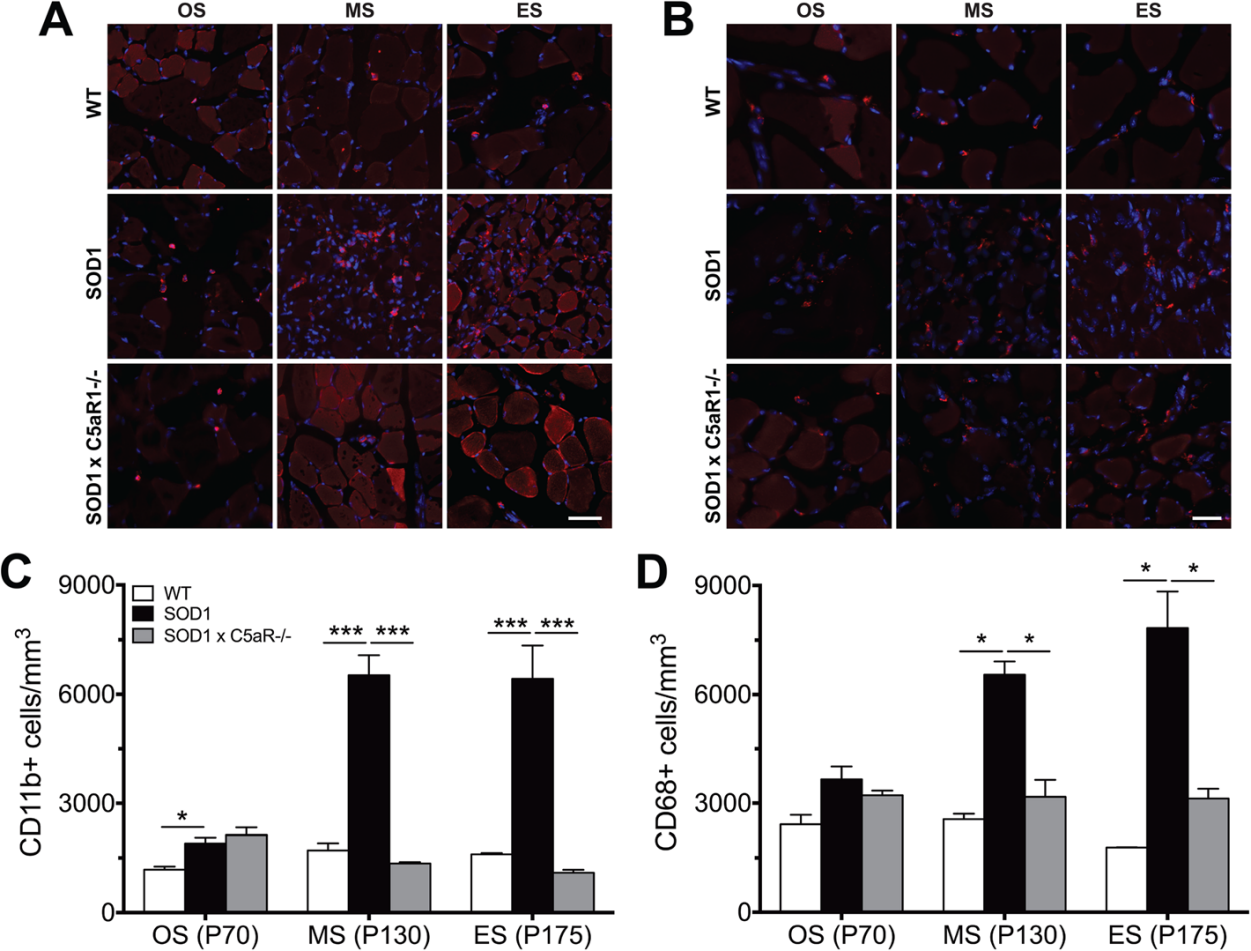
Reduction of CD11b^+^/CD68^+^ macrophages in tibialis anterior (TA) muscle of hSOD1^G93A^ mice lacking C5aR1 (SOD1 × C5aR1^-/-^) when compared to hSOD1^G93A^ (SOD1) mice. TA muscle from wild-type (WT), SOD1 and SOD1 × C5aR1^-/-^ mice were stained for CD11b and CD68 for macrophages. **a** and **b** Representative images of CD11b and CD68 in WT, SOD1 and SOD1 x C5aR1^-/-^ mice at onset, mid-symptomatic and end-stage of disease. Scale bar = 20 μm. **c** More CD11b^+^ macrophages were present in SOD1 mice at onset (OS; P70), mid-symptomatic (MS; P130) and end-stage of disease (ES; P175) when compared to WT mice, while a reduction in CD11b^+^ macrophages were present in SOD1 × C5aR1^-/-^ mice at MS and ES when compared with SOD1 mice (*n* = 3; * *p* < 0.05, *** *p* < 0.001, one-way ANOVA with Tukey’s *post hoc* test at each stage of disease). **d** CD68^+^ macrophages were also increased in SOD1 mice compared to WT mice at MS and ES with a decrease in SOD1 × C5aR1^-/-^ at these stages (*n* = 3, * *p* < 0.05, one-way ANOVA with Tukey’s *post hoc* test at each stage of disease). Data in **c** and **d** are expressed as means ± SEM

The number of CD11b^+^ macrophages in TA muscle of hSOD1^G93A^ mice significantly increased at onset, mid-symptomatic and end-stage of disease when compared to WT mice (*n* = 3, * *p* < 0.05 and *** *p* < 0.001; Fig. 5a, c white and black bars). Interestingly the number of CD11b^+^ macrophages in TA muscle of hSOD1^G93A^ mice lacking C5aR1 significantly reduced at mid-symptomatic and end-stage of disease when compared to hSOD1^G93A^ mice (*n* = 3, *** *p* < 0.001; Fig. 5a, c black and grey bars). Similarly, the number of CD68^+^ macrophages in TA muscle of hSOD1^G93A^ mice significantly increased at mid-symptomatic and end-stage of disease when compared to WT mice (*n* = 3, * *p* < 0.05; Fig. 5b, d white and black bars). Again, a significant decrease in the number of CD68^+^ macrophages in the TA muscle of hSOD1^G93A^ mice lacking C5aR1 at mid-symptomatic and end-stage of disease when compared to hSOD1^G93A^ mice (*n* = 3, *p < 0.05; Fig. 5b, d, black and grey bars). This demonstrates that C5a-C5aR1 signalling induces the infiltration of the peripheral monocytes/macrophages in hSOD1^G93A^ mice, which may potentially affect the progression of denervation in these muscles. As a further control, the numbers of macrophages were also quantified in healthy C5aR1-deficient mice in TA muscle at all stages, which showed no significant difference to WT mice (*data not shown*).

### hSOD1^G93A^ mice lacking C5aR1 have reduced denervation of NMJ with improvement in hind-limb grip strength when compared to hSOD1^G93A^ mice

**Fig. 6:**
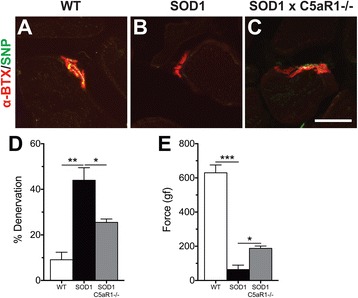
Ablation of C5a-C5aR1 signalling in hSOD1^G93A^ (SOD1) mice reduces neuromuscular (NMJ) denervation and improves hind-limb grip strength. Panels **a** to **c** shows double immuno-labelling of neuromuscular junction ($\boldsymbol{\upalpha}$BTX; *red*) with pre-synaptic marker (SNP; *green*) for WT (**a**), SOD1 (**b**) and SOD1 x C5aR1^-/-^ (**c**) mice at mid-symptomatic stage of disease. **d** SOD1 x C5aR1^-/-^ mice showed reduction in NMJ denervation when compared to SOD1 mice at mid-symptomatic stage of disease (*n* = 3, * *p* < 0.05 and ** *p* < 0.01, one-way ANOVA with Tukey’s *post hoc* test). **e** This was further supported by hind-limb grip strength, where SOD1 x C5aR1^-/-^ mice showed improvement in motor deficits when compared to SOD1 mice at mid-symptomatic stage of disease (*n* = 10, * *p* < 0.05 and *** *p* < 0.001, one-way ANOVA with Tukey’s *post hoc* test). Data in **d** and **e** are expressed as means ± SEM. Scale bar = 20 μm

In addition to reduction in macrophages in the TA muscle of hSOD1^G93A^ mice lacking C5aR1, we next investigated if ablation of C5a – C5aR1 signalling could improve motor function in these animals by measuring denervation status and hind-limb grip strength at mid-symptomatic stage. hSOD1^G93A^ mice lacking C5aR1 showed reduced proportion of denervated NMJs when compared to hSOD1^G93A^ mice (hSOD1^G93A^ mice = 43.96 ± 5.51% (83 NMJs) vs hSOD1^G93A^ × C5aR1^-/-^ mice = 25.49 ± 1.50% (98 NMJs), *n* = 3, * *p* < 0.05 and ** *p* < 0.01; Fig. 6a–d). To further support this improvement in muscle denervation, motor deficits were also assessed in these animals using hind-limb grip strength, a sensitive marker of neuromotor performance (Lee et al., 2017). Ablation of C5aR1 in hSOD1^G93A^ mice counteracted the loss of hind-limb grip strength at mid-symptomatic age (*n* = 10, * *p* < 0.05 and *** *p* < 0.001; Fig. 6e).

## Discussion

The major findings of the current study are that in the TA muscle of hSOD1^G93A^ mice, the terminal complement system (C5a and C5aR1) is up-regulated, and the complement receptor C5aR1 is responsible for the recruitment of peripheral macrophages during ALS disease progression. It has been well documented that the complement cascade may be involved in the disease progression of ALS, with evidence from both human patients and rodent models [7]. The present study adds to this knowledge, demonstrating the up-regulation of mRNA expression in C1qB, C4, factor B, C3, and regulators CD55 and CD59a, in the TA muscles of hSOD1^G93A^ mice. These results mirror our previous demonstration of complement component up-regulation in the spinal cord of hSOD1^G93A^ mice [8]. This local terminal complement signalling in the skeletal muscle might therefore contribute to the denervation of neuromuscular synapses in hSOD1^G93A^ mice by promoting infiltration of peripheral macrophages through C5a-C5aR1 signalling, possibly to phagocytose/repair the degenerating neuromuscular synapses during disease progression.

The present study provided evidence for the dysregulation of classical (C1qB), lectin (C4), and alternate (factor B) pathways of the complement system in the TA muscle of hSOD1^G93A^ mice during ALS disease progression. This is consistent with numerous studies in mouse models of ALS where deposition of C3 activation products and C1q were present in the hSOD1^G93A^ mice motor endplate [10, 17]. This may suggest that up-regulation of complement components could assist in the removal of degenerating neuromuscular synapses via phagocytosis, during disease progression in hSOD1^G93A^ mice [17]. Among the complement activation effector molecules, C5a is considered the most potent peptide, and it contains a broad range of biological functions that may contribute to its deleterious effects during pathology [26]. C5a exerts its major effects through its predominantly expressed receptor, C5aR1 [7]. Previous studies have demonstrated up-regulation of C5aR1 within the CNS of hSOD1^G93A^ rats and mice, as well as in human ALS patients, suggesting that heightened C5a-C5aR1 signalling plays a role in the ALS pathology [8, 11, 12]. In the present study, we demonstrated that the expression of C5aR1 in hSOD1^G93A^ TA muscle is elevated at both mRNA and protein levels. In addition, C5a, the ligand of C5aR1, is also increased, confirming terminal pathway complement activation in these muscles. This suggests that enhanced C5a – C5aR1 signalling may affect the disease progression of ALS, in part through actions within the skeletal muscle.

To further investigate the role of C5aR1, we examined the cellular localisation of C5aR1 in skeletal muscle at motor endplates and macrophages. We found no evidence for expression of C5aR1 at motor endplate or Schwann cells. This was intriguing as this was not consistent with previous study which showed evidence of membrane attack complex (MAC) part of the terminal pathway in the neuromuscular junction of hSOD1^G93A^ mice [27]. However, as the function of C5aR1 and MAC is physiologically different [28], it is plausible to suggest that they could induce neuromuscular junction denervation through different pathways; namely C5b could associate with other component of MAC at degenerating NMJs directly, while C5a would act as a chemoatrractant and work via C5aR1 at sites close to the NMJ in the skeletal muscle of hSOD1^G93A^ mice.

In support of this, we identified C5aR1 expression on macrophages within both WT and hSOD1^G93A^ TA muscles. Skeletal muscle macrophages are cells that are primarily involved in muscle regeneration and repair during skeletal muscle injury, also termed as tissue healing macrophages, however chronic accumulation of macrophages can switch them to classical inflammatory macrophages, which can exacerbate damage to the skeletal muscle [29]. Macrophages are known to express C5aR1 [30], and our findings in TA muscles support this. In addition, macrophages are also known to infiltrate and accumulate in hSOD1^G93A^ mice muscles [10]. In concordance with this, the present study found a high degree of macrophage infiltration in TA muscles of hSOD1^G93A^ mice, beginning from disease onset age. Remarkably, this infiltration was almost completely attenuated in hSOD1^G93A^ mice lacking C5aR1, demonstrating that C5a is the predominant skeletal muscle recruiter of these immune cells in hSOD1^G93A^ mice. This may suggest that absence of C5a-C5aR1 signalling could slow ALS disease progression [11–13] by reducing the number of pro-inflammatory macrophages infiltrating the TA muscle in hSOD1^G93A^ mice, ultimately reducing the self-damage to neuromuscular junctions. However, as this study utilised full knockout of C5aR1 in all tissues, future investigation using conditional knockout mice [30] or bone-marrow chimeras [31] to delete C5aR1 either peripherally or centrally, could help us and fellow researchers determine if C5a-C5aR1 signalling in the CNS or TA muscle (or both) affects ALS disease progression.

## Conclusion

In summary, this study presents evidence that the terminal innate immune complement system is activated in skeletal muscles of hSOD1^G93A^ mice, and may contribute to motor deficits through the recruitment of activated macrophages. This improved understanding of the mechanisms by which C5aR1 contributes to ALS disease, may help guide future therapeutic interventions targeting complement signalling.

## Abbreviations

αBTX: Alpha-Bungaratoxin
ALS: Amyotrophic lateral sclerosis
CNS: Central nervous system
DAPI: Diamidino-2-phenylindole
TA: Tibialis anterior
WT: Wild-type

## References

[1] Hooten KG, Beers DR, Zhao W, Appel SH (2015). Protective and Toxic Neuroinflammation in Amyotrophic Lateral Sclerosis. Neurotherapeutics.

[2] Beers DR, Henkel JS, Zhao W, Wang J, Appel SH (2008). CD4+ T cells support glial neuroprotection, slow disease progression, and modify glial morphology in an animal model of inherited ALS. Proc Natl Acad Sci U S A.

[3] Butovsky O, Jedrychowski MP, Cialic R, Krasemann S, Murugaiyan G, Fanek Z, Greco DJ, Wu PM, Doykan CE, Kiner O (2015). Targeting miR-155 restores abnormal microglia and attenuates disease in SOD1 mice. Ann Neurol.

[4] Butovsky O, Siddiqui S, Gabriely G, Lanser AJ, Dake B, Murugaiyan G, Doykan CE, Wu PM, Gali RR, Iyer LK (2012). Modulating inflammatory monocytes with a unique microRNA gene signature ameliorates murine ALS. J Clin Invest.

[5] Henkel JS, Beers DR, Wen S, Rivera AL, Toennis KM, Appel JE, Zhao W, Moore DH, Powell SZ, Appel SH (2013). Regulatory T-lymphocytes mediate amyotrophic lateral sclerosis progression and survival. EMBO Mol Med.

[6] Zhao W, Beers DR, Liao B, Henkel JS, Appel SH (2012). Regulatory T lymphocytes from ALS mice suppress microglia and effector T lymphocytes through different cytokine-mediated mechanisms. Neurobiol Dis.

[7] Brennan FH, Lee JD, Ruitenberg MJ, Woodruff TM (2016). Therapeutic targeting of complement to modify disease course and improve outcomes in neurological conditions. Semin Immunol.

[8] Lee JD, Kamaruzaman NA, Fung JN, Taylor SM, Turner BJ, Atkin JD, Woodruff TM, Noakes PG (2013). Dysregulation of the complement cascade in the hSOD1G93A transgenic mouse model of amyotrophic lateral sclerosis. J Neuroinflammation.

[9] Lobsiger CS, Boillee S, Pozniak C, Khan AM, McAlonis-Downes M, Lewcock JW, Cleveland DW (2013). C1q induction and global complement pathway activation do not contribute to ALS toxicity in mutant SOD1 mice. Proc Natl Acad Sci U S A.

[10] Chiu IM, Phatnani H, Kuligowski M, Tapia JC, Carrasco MA, Zhang M, Maniatis T, Carroll MC (2009). Activation of innate and humoral immunity in the peripheral nervous system of ALS transgenic mice. Proc Natl Acad Sci U S A.

[11] Lee JD, Kumar V, Fung JN, Ruitenberg MJ, Noakes PG, Woodruff TM. Pharmacological inhibition of complement C5a-C5aR1 signalling ameliorates disease pathology in the hSOD1G93A mouse model of amyotrophic lateral sclerosis. Br J Pharmacol. 2017. 10.1111/bph.13730 PMC5368046 28128456

[12] Woodruff TM, Costantini KJ, Crane JW, Atkin JD, Monk PN, Taylor SM, Noakes PG (2008). The complement factor C5a contributes to pathology in a rat model of amyotrophic lateral sclerosis. J Immunol.

[13] Woodruff TM, Lee JD, Noakes PG (2014). Role for terminal complement activation in amyotrophic lateral sclerosis disease progression. Proc Natl Acad Sci U S A.

[14] Aggarwal A, Nicholson G (2002). Detection of preclinical motor neurone loss in SOD1 mutation carriers using motor unit number estimation. J Neurol Neurosurg Psychiatry.

[15] Fischer LR, Culver DG, Tennant P, Davis AA, Wang M, Castellano-Sanchez A, Khan J, Polak MA, Glass JD (2004). Amyotrophic lateral sclerosis is a distal axonopathy: evidence in mice and man. Exp Neurol.

[16] de Jonge RR, van Schaik IN, Vreijling JP, Troost D, Baas F (2004). Expression of complement components in the peripheral nervous system. Hum Mol Genet.

[17] Heurich B, El Idrissi NB, Donev RM, Petri S, Claus P, Neal J, Morgan BP, Ramaglia V (2011). Complement upregulation and activation on motor neurons and neuromuscular junction in the SOD1 G93A mouse model of familial amyotrophic lateral sclerosis. J Neuroimmunol.

[18] Rosen DR, Siddique T, Patterson D, Figlewicz DA, Sapp P, Hentati A, Donaldson D, Goto J, O'Regan JP, Deng HX (1993). Mutations in Cu/Zn superoxide dismutase gene are associated with familial amyotrophic lateral sclerosis. Nature.

[19] Hollmann TJ, Mueller-Ortiz SL, Braun MC, Wetsel RA (2008). Disruption of the C5a receptor gene increases resistance to acute Gram-negative bacteremia and endotoxic shock: opposing roles of C3a and C5a. Mol Immunol.

[20] Livak KJ, Schmittgen TD (2001). Analysis of relative gene expression data using real-time quantitative PCR and the 2(-Delta Delta C(T)) Method. Methods.

[21] Atkin JD, Scott RL, West JM, Lopes E, Quah AK, Cheema SS (2005). Properties of slow- and fast-twitch muscle fibres in a mouse model of amyotrophic lateral sclerosis. Neuromuscul Disord.

[22] Frey D, Schneider C, Xu L, Borg J, Spooren W, Caroni P (2000). Early and selective loss of neuromuscular synapse subtypes with low sprouting competence in motoneuron diseases. J Neurosci.

[23] Woodruff TM, Ager RR, Tenner AJ, Noakes PG, Taylor SM (2010). The role of the complement system and the activation fragment C5a in the central nervous system. Neuromolecular Med.

[24] Humayun S, Gohar M, Volkening K, Moisse K, Leystra-Lantz C, Mepham J, McLean J, Strong MJ (2009). The complement factor C5a receptor is upregulated in NFL-/- mouse motor neurons. J Neuroimmunol.

[25] Marder SR, Chenoweth DE, Goldstein IM, Perez HD (1985). Chemotactic responses of human peripheral blood monocytes to the complement-derived peptides C5a and C5a des Arg. J Immunol.

[26] Lee JD, Lee JY, Taylor SM, Noakes PG, Woodruff TM. Innate Immunity in ALS. In Amyotrophic Lateral Sclerosis. Edited by Maurer MH: InTech; 2012

[27] Bahia El Idrissi N, Bosch S, Ramaglia V, Aronica E, Baas F, Troost D. Complement activation at the motor end-plates in amyotrophic lateral sclerosis. J Neuroinflammation. 2016;13:72. 10.1186/s12974-016-0538-2 PMC4823861 27056040

[28] Noris M, Remuzzi G (2013). Overview of complement activation and regulation. Semin Nephrol.

[29] Bosurgi L, Manfredi AA, Rovere-Querini P (2011). Macrophages in injured skeletal muscle: a perpetuum mobile causing and limiting fibrosis, prompting or restricting resolution and regeneration. Front Immunol.

[30] Karsten CM, Laumonnier Y, Eurich B, Ender F, Broker K, Roy S, Czabanska A, Vollbrandt T, Figge J, Kohl J (2015). Monitoring and cell-specific deletion of C5aR1 using a novel floxed GFP-C5aR1 reporter knock-in mouse. J Immunol.

[31] Wu MC, Brennan FH, Lynch JP, Mantovani S, Phipps S, Wetsel RA, Ruitenberg MJ, Taylor SM, Woodruff TM (2013). The receptor for complement component C3a mediates protection from intestinal ischemia-reperfusion injuries by inhibiting neutrophil mobilization. Proc Natl Acad Sci U S A.

